# Microsatellite instability testing in colorectal cancer using the QiaXcel advanced platform

**DOI:** 10.1186/s12885-018-4400-z

**Published:** 2018-04-27

**Authors:** Isabel Förster, Michael Brockmann, Oliver Schildgen, Verena Schildgen

**Affiliations:** Kliniken der Stadt Köln gGmbH, Institut für Pathologie, Klinikum der Privaten Universität Witten/Herdecke mit Sitz in Köln, Ostmerheimer Str. 200, D-51109 Köln Cologne, Germany

**Keywords:** Colo-rectal cancer, Microsatellites, MSI, QiaXcel advanced

## Abstract

**Background:**

Microsatellite instability (MSI) is a major predictive and diagnostic marker in several cancers including colorectal carcinomas. Diagnostic testing for microsatellites is generally performed using capillary sequencers, which requires expensive high-end equipment including expensive chemistry using fluorescent dyes labelling the PCR products of interest. In this study we have modified such a diagnostic protocol and established the microsatellite testing on the QiaXcel Advanced platform.

**Methods:**

MSI testing was based on a previously established protocol describing a multiplex PCR followed by fluorescent detection of PCR products in a capillary sequencing device. Ten microsatellites were included in the new protocol: BAT25, BAT26, BAT40, D2s123, D10s197, D13s153, D17s250, D18s58, D5s346, and MycI. In this protocol the PCR was demultiplexed and established on the QiaXcel Advanced system (Qiagen, Hilden, Germany).

**Results:**

Making use of a series of FFPE control samples with known MSI status including those with and without MSI a protocol for MSI testing was successfully established on the QiaXcel Advanced platform.

**Conclusions:**

MSI testing for human colorectal cancers using the QiaXcel Advanced system could serve as an economic acceptable tool for rapid diagnostics in laboratories that do not have access to a capillary sequencing unit.

## Background

Microsatellites are non-coding DNA sequences that occur ubiquitous in all eukaryotic genomes and are a powerful tool for the analysis of populations, genetic diversity, and paternity tests [1]. The analysis of microsatellites therefore is used in many disciplines, including botany, genetics, zoology, medical microbiology, and others [1–9].

Also pathologists and oncologists have become aware of microsatellites, as microsatellite instabilities (MSI) frequently occur in several human cancers, mainly in colorectal carcinomas. These instabilities indicate that the mismatch repair system of the host cells is likely damaged and can serve as a predictive and diagnostic marker [10–14].

As early as in 2005 Popat and coworkers have published a systematic review of the instability of microsatellites and their usage as markers in the prognosis of colorectal cancers [15]. The authors have concluded that patients with MSI have a significantly better prognosis than MS-stable tumors and had a better response to chemotherapy (reviewed by [16]). Meanwhile it has been shown that also other tumor types could be associated to MSI [17]. In particular, 14 of 18 cancers, such as endometrial, gastric, and colon cancer had high percentages of MSI. The study by Hause and colleagues [17] also revealed that MSI testing can be used to classify tumor types on a molecular level in four different groups named A-D; e.g. colon and rectal cancers clustered in group A, whereas liver hepatocellular carcinomas and kidney renal carcinomas clustered in group D. These two studies are examples that clearly show that MSI testing is a useful tool to determine the molecular tumor type and enables the pathologist to make a better prognosis for the subsequent chemo-therapy.

So far the most requested MSI testing in colorectal cancers appears to be the most relevant testing in Germany and since a few years there is also a round robin trial organized by German pathologists. However, diagnostic testing for microsatellites generally requires high-end laboratory equipment in the form of a capillary sequencing device capable to distinguish between multiple fluorescent dyes, thus the MSI testing is limited to highly specialized laboratories. A more broadly available device are capillary electrophoresis systems that are frequently used in diagnostic laboratories for quality analyses of DNA and RNA isolated from clinical specimen including formalin fixed paraffin embedded (FFPE) tissues. The aim of this study was to establish a protocol for MSI testing on the QiaXcel Advanced system (Qiagen, Hilden, Germany). It was previously shown that this system is feasible for plant genotyping via microsatellite analyses [1] and can be used for MSI testing in human endometric cancers [18], but was not yet used for the analysis of colorectal tumors.

## Methods

DNA used for this study was obtained from clinical samples previously tested positive for MSI by immunohistochemistry for mismatch repair defects. Unfortunately, as these samples were tested externally for MSI by IHC, no more details on the results of MSI-IHC testing were available. A collection of 5 samples with MSI and 5 control samples without MSI could be included in this pilot study. In all ten cases healthy tissues of the respective patients were use as controls. DNA was extracted using Maxwell FFPE DNA extraction kits (Promega, Mannheim, Germany). In addition, we have included 8 samples from a recent German round robin trial for HPNCC MSI (http://www.quip-ringversuche.de/pdf/2017/QuIP-Programm-2017.pdf; last page view 16th April 2018). For these samples we have received the results for MSI testing for markers of the Bethesda panel and the information if the samples had a MSI-high, MSI-low or MS-stable status.

For this study a previously established MSI detection protocol that included the following microsatellites has been de-multiplexed (Table 1). This primer set includes the recommend Bethesda protocol primers [19] plus 5 additional markers that could be useful for MSI detection [20, 21]; this latter extended panel was shown to be useful to confirm the MSS status of hereditary colorectal carcinoma not caused by common mutations in the mismatch repair genes [21]. According to the Bethesda agreement a sample is considered as MSI-H if two or more markers of the Bethesda panel are mutated. Thereby, the additional markers were recommended by Wolfgang Dietmaier (University Hospital Regensburg, personal communication) who organized the last German round robin trial for testing of MSI in hereditary colorectal cancer. The additional markers serve as back-up if one of the Bethesda panel markers cannot be properly determined due to technical reasons such as PCR inhibition or DNA fragmentation.

**Table 1:** Forward and reverse primer used for the detection of the respective microsatellites

Microsatellite	Forward-primer	Reverse-primer
BAT 25	TCGGCTCCAAGAATGTAAGT	TCTGCATTTTAACTATGGCTC
BAT 26	TGACTACTTTTGACTTCAGCC	AACCATTCAACATTTTTAACCC
BAT 40	GTAGAGCAAGACCACCTTG	ATTAACTTCCTACACCACAAC
D2s123	AAACAGGATGCCTGCCTTTA	GGACTTTCCACCTATGGGAC
D10s197	GTGATACTGTCCTCAGGTCTCC	ACCACTGCACTTCAGGTGAC
D13s153	AGCATTGTTTCATGTTGGTG	CAGCAGTGAAGGTCTAAGCC
D17s250	GGAAGAATCAAATAGACAAT	GCTGGCCATATATATATTTAAACC
D18s58	GCAGGAAATCGCAGGAACTT	GCTCCCGGCTGGTTTT
D5s346	ACTCACTCTAGTGATAAATCG	AGCAGATAAGACAGTATTACTAGTT
MycI	CCTTTTAAGCTGCAACAATTTC	TGGCGAGACTCCATCAAAG

For the PCR the HotStar Taq PCR Mastermix Kit (Qiagen, Hilden Germany) was used according to the manufacturer’s recommendation with respectively 1 μl forward and reverse primer (10 pmol/μl each). The final volume of each PCR was 25 μl including 5 μl from the extracted DNA (1 ng/μl). Detection and differentiation of PCR fragments were performed on the QiaXcel Advanced System using a run method based on the instrument setting OM500, AM 15 bp–600 bp, and SM 25-500 bp.

## Results

Microsatellite testing was performed in a set of clinical controls and round robin trial specimens that were previously tested positive for MSI by immunohistochemistry (IHC) and confirmed in an external laboratory by fluorescent dye based capillary electrophoresis..Based on these specimen cohort PCRs were performed for the 10 microsatellites BAT25, BAT26, BAT40, D2s123, D10s197, D13s153, D17s250, D18s58, D5s346, and MycI, of which BAT25, BAT26, D5s346, D17s250 and D2s123 originate from the Bethesda panel [10, 12, 16, 22]. In total 20 PCRs were performed per patient, as for every microsatellite tumor DNA was compared to DNA from the healthy control tissue, respectively.

**Fig. 1:**
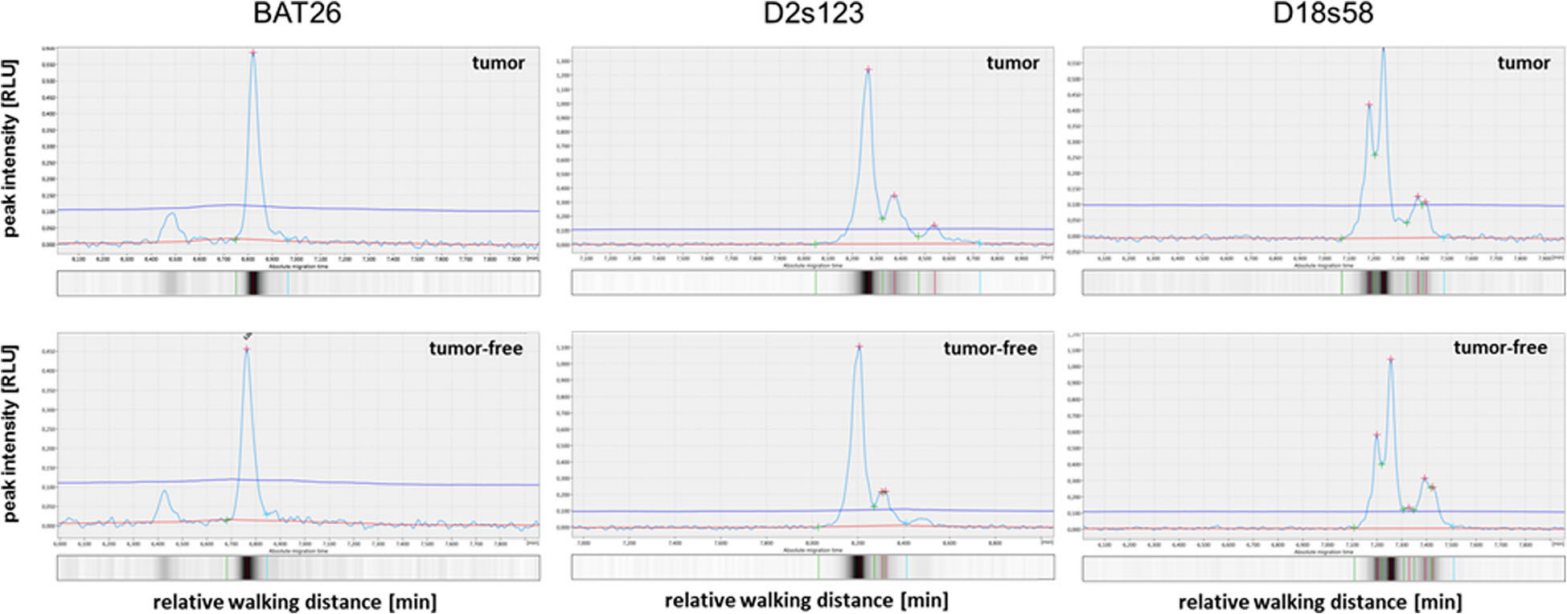
Electropherograms and virtual gel lanes obtained for BAT26, D2s123, and D18s58. The figure shows tumor (top) vs. tumor-free tissue (bottom) of different patients with stable microsatellite status (wild-type)

In case of stable microsatellites, the electropherogram shows the same pattern in healthy tissue as well as in the tumor, but may vary in the overall intensity (Fig. 1).

**Fig. 2:**
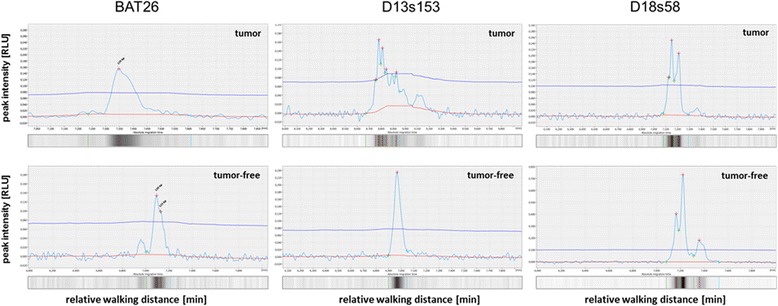
Electropherograms and virtual gel lanes obtained for BAT26, D13s153, and D18s58. The panel shows tumor (top) vs. tumor-free tissue (bottom) of different Patients with unstable microsatellites (MSI)

**Fig. 3:**
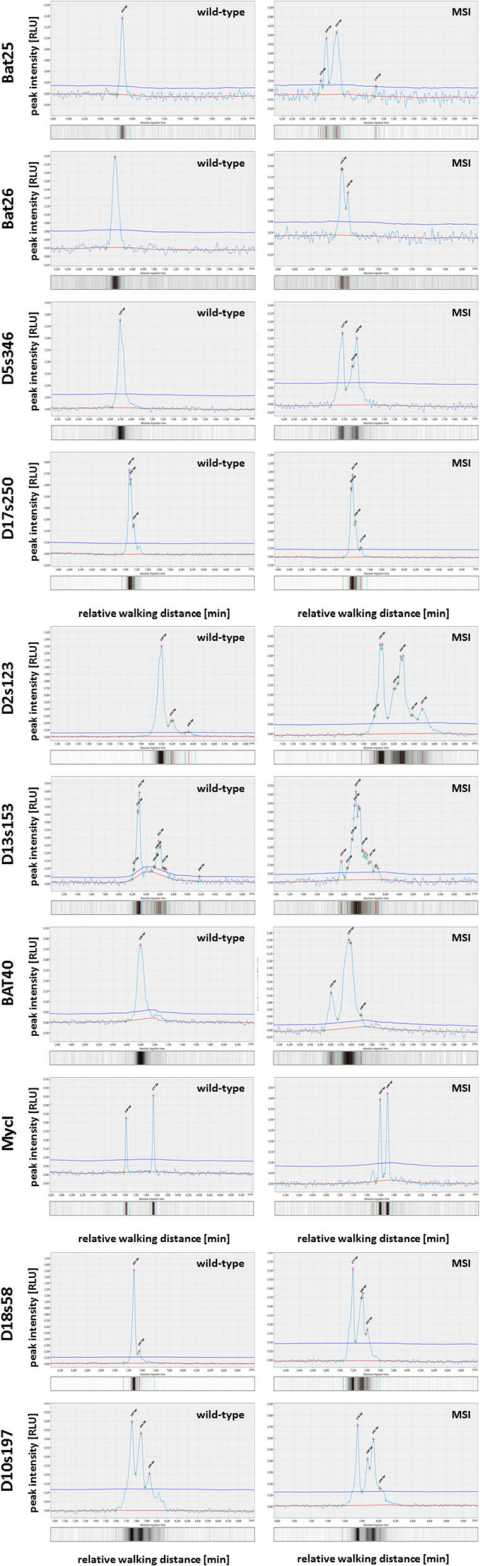
Electropherograms and virtual gel lanes exemplarily shown for all 10 microsatellites included in the protocol. For all microsatellites a tumor free specimen versus a tumor tissue is shown, thereby tumor and tumor free peaks shown for the respective marker originate from the same donor. The samples used in our study were considered positive if the peak pattern differed between tumor and tumor free tissue. Of note, we observed that the marker D17s250 may only differ slightly in the first peak in which a shift in the first double peak was observed that was less extensive as observed for the other markers. This phenomenon is visible if the figure is enlarged

Unstable microsatellites have been identified in all previous MSI positive tested samples with the QiaXcel system in direct comparison between healthy tissue and tumor as exemplarily shown for BAT26, D13s153, D18s58 (Fig. 2). Figure 3 shows the comparison of healthy versus tumor tissue for all 10 microsatellite markers included in our protocol. In most cases the differences are obvious and result in a different peak pattern in case of the instabilities, mostly characterized by additional peaks or a peak-shift to the right side of the diagram (which in turn indicates longer MS-sequences).

The re-testing of round robin trial samples revealed a full match between the round robin results that were generated with a capillary sequencing unit and the protocol established for the present study (Table 2). Thereby, the round robin trial was designed according to the Bethesda panel and it was not discriminated between MSI-low (MSI-L) and MS-stable (MSS). However, all unstable tumors were MSI-high (MSI-H) in the round robin samples.

**Table 2:** Results obtained from samples of the round robin test. Reference values were obtained from the trial coordinators by a multiplex assay analyzed on a capillary sequencer and reported to our laboratory. Afterwards, the samples were re-analyzed on the QiaXcel Advanced system. All samples could be fully confirmed by analyzing the peak patterns

Round Robin Sample		BAT25	BAT26	D5s346	D2S123	D17s250	MS-status
1	expected	wt	wt	wt	wt	wt	MSS/MSI-L
	our result	wt	wt	wt	wt	wt	MSS
2	expected	MSI	MSI	MSI	MSI	MSI	MSI-H
	our result	MSI	MSI	MSI	MSI	MSI	MSI-H
3	expected	wt	wt	wt	wt	wt	MSS/MSI-L
	our result	wt	wt	wt	wt	wt	MSS
4	expected	MSI	MSI	MSI	MSI	MSI	MSI-H
	our result	MSI	MSI	MSI	MSI	MSI	MSI-H
5	expected	wt	wt	wt	wt	wt	MSS/MSI-L
	our result	wt	wt	wt	wt	wt	MSS
6	expected	wt	wt	wt	wt	wt	MSS/MSI-L
	our result	wt	wt	wt	wt	wt	MSS
7	expected	MSI	MSI	MSI	MSI	MSI	MSI-H
	our result	MSI	MSI	MSI	MSI	MSI	MSI-H
8	expected	MSI	MSI	MSI	MSI	MSI	MSI-H
	our result	MSI	MSI	MSI	MSI	MSI	MSI-H

## Discussion

The QiaXcel system was previously used for microsatellite analyses for plant species and for endometrial cancer [1, 18], but so far has not been used for colorectal cancer typing. In our pretested clinical pilot cohort the QiaXcel advanced system confirmed the previous IHC results of all samples. Because of the small patient cohort we were not able to detect instabilities for BAT25, D10s197, D17s250 and MycI, but were able to detect those instabilities in a further set of samples originating from the last round robin trial we participated (examples shown in Fig. 3). Our results show, that this typing is also possible with the QiaXcel system and it thus may be used in those pathology institute that have not yet established MSI-testing on other platforms or lack a capillary sequencing device.

For the estimation of the assay costs, the reagents used for the proposed method and for usage of a capillary sequencer-based protocol were taken into account. For capillary electrophoresis dye-labeled primers, the capillary usage itself, and the list price for multiplex-suitable PCR chemistry was calculated. Per patient two reactions would be required for the tumor tissue and two for the tumor free tissue, resulting in approx. 17–18 € per patient. The QiaXcel capillary unit costs approx. 750 € plus PCR reagents, and has 104 runs with 12 lanes per run. With the proposed protocol 10 lanes are required for the tumor free tissue, and 10 lanes are used for the tumor tissue, plus an additional lane for the size marker, resulting also in 17–18 € per patient analysis. The “hardware” costs, however, differ significantly. The German list price for the QiaXcel Advanced system is approx. 29.000 €, whereas the list price for a capillary sequencing unit is between 60.000 and 120.000 €.

The only experienced disadvantage during the setup of the MSI testing protocol was that de-multiplexing rapidly leads to large experimental approaches of 20 PCRs per patient being error-prone in routine settings with large numbers of samples. To reduce the amount of single PCRs it would be possible to combine such targets with distinct difference in size. The fact that microsatellites vary among individuals is technically used for phylogenetic analyses such as parental testing [7, 8], but implicates for colorectal cancer testing that for each patient corresponding tumor-free tissue controls are required. And it must be taken into account that variances in the overall intensity of the electropherograms most likely result from DNA quality and DNA preparation from FFPE material.

## Conclusion

In summary, we come to the conclusion that the QiaXcel system is appropriate for validation for routine diagnostics of MSI testing in colorectal cancers and routine usage in pathology laboratory without a capillary sequencing device available.

## Abbreviations

FFPE: formalin fixed paraffin embedded
IHC: immunohistochemistry
MSI: Microsatellite instability
